# Supplementary material for: Bird population declines and species turnover are changing the acoustic properties of spring soundscapes
Source: Nat Commun. 2021 Nov 2;12:6217. doi: 10.1038/s41467-021-26488-1 (PMC8564540; doi:10.1038/s41467-021-26488-1)
Supplement: Supplementary file 1 — Supplementary Information File [file 41467_2021_26488_MOESM1_ESM.pdf]

Bird population declines and species turnover are changing the acoustic properties of spring soundscapes

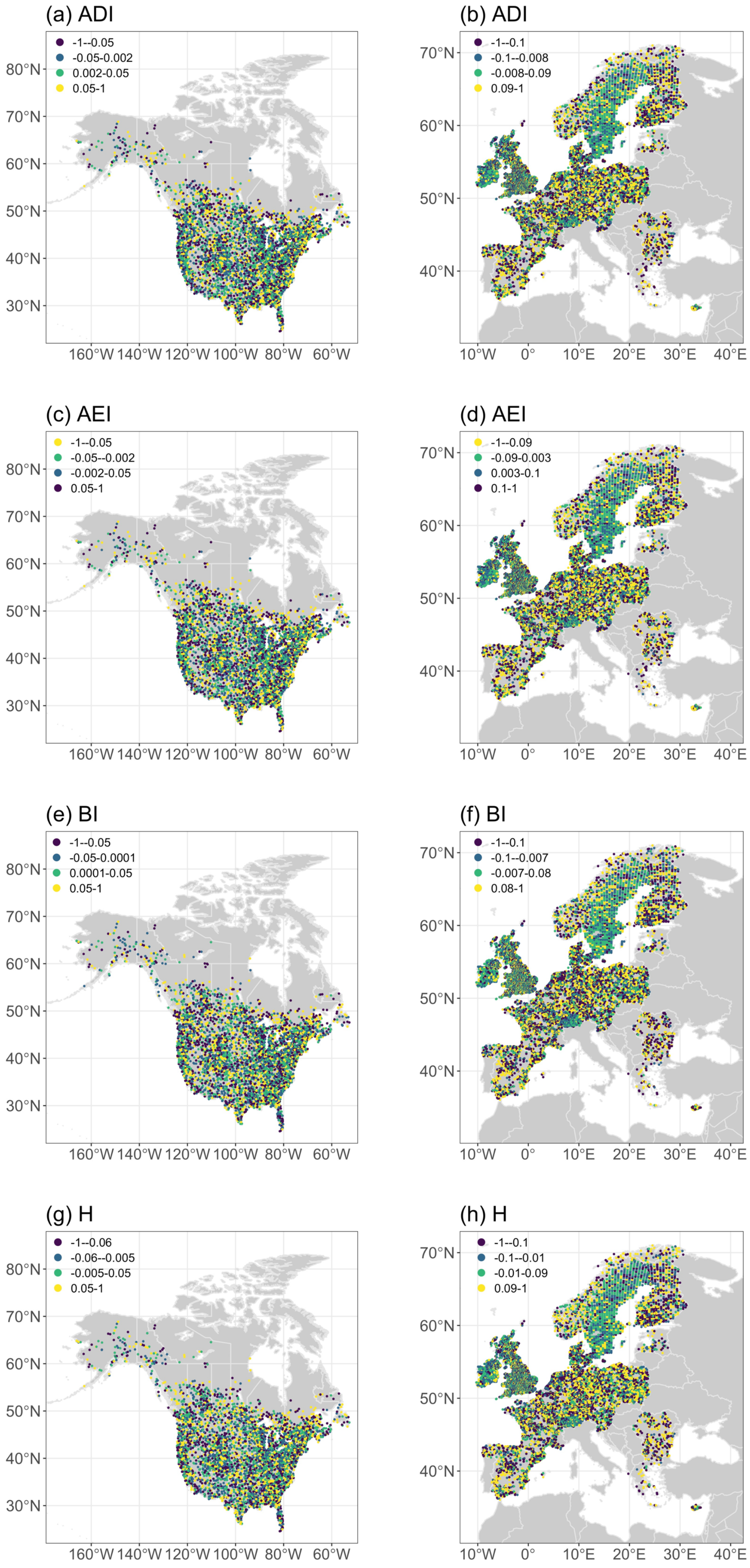
(a) ADI
-1--0.05
-0.05-0.002
0.002-0.05
0.05-1
80°N
70°N
60°N
50°N
40°N
30°N
160°W 140°W 120°W 100°W 80°W 60°W
(b) ADI
-1--0.1
-0.1--0.008
-0.008-0.09
0.09-1
70°N
60°N
50°N
40°N
10°W 0° 10°E 20°E 30°E 40°E
(c) AEI
-1--0.05
-0.05--0.002
-0.002-0.05
0.05-1
80°N
70°N
60°N
50°N
40°N
30°N
160°W 140°W 120°W 100°W 80°W 60°W
(d) AEI
-1--0.09
-0.09-0.003
0.003-0.1
0.1-1
70°N
60°N
50°N
40°N
10°W 0° 10°E 20°E 30°E 40°E
(e) BI
-1--0.05
-0.05-0.0001
0.0001-0.05
0.05-1
80°N
70°N
60°N
50°N
40°N
30°N
160°W 140°W 120°W 100°W 80°W 60°W
(f) BI
-1--0.1
-0.1--0.007
-0.007-0.08
0.08-1
70°N
60°N
50°N
40°N
10°W 0° 10°E 20°E 30°E 40°E
(g) H
-1--0.06
-0.06--0.005
-0.005-0.05
0.05-1
80°N
70°N
60°N
50°N
40°N
30°N
160°W 140°W 120°W 100°W 80°W 60°W
(h) H
-1--0.1
-0.1--0.01
-0.01-0.09
0.09-1
70°N
60°N
50°N
40°N
10°W 0° 10°E 20°E 30°E 40°E

Supplementary Figure 1: Site-level spatial variation in acoustic index trends. Site-level trends in Acoustic Diversity Index, ADI (a,b), Acoustic Evenness Index, AEI (c,d), Bioacoustic Index, BI (e,f) and Acoustic Entropy, H (g,h) at the first site of 4196 NA-BBS routes across North America between 1996 and 2017 and in 16524 PECBMS sites across Europe surveyed in at least three years between 1998 and 2018. Colours indicate the size and direction of trend; note that the colour scheme is reversed for AEI, as positive trends are taken to represent a reduction in soundscape quality for this metric. Note that site-level trends are derived from changes in standardised annual values of each acoustic index.

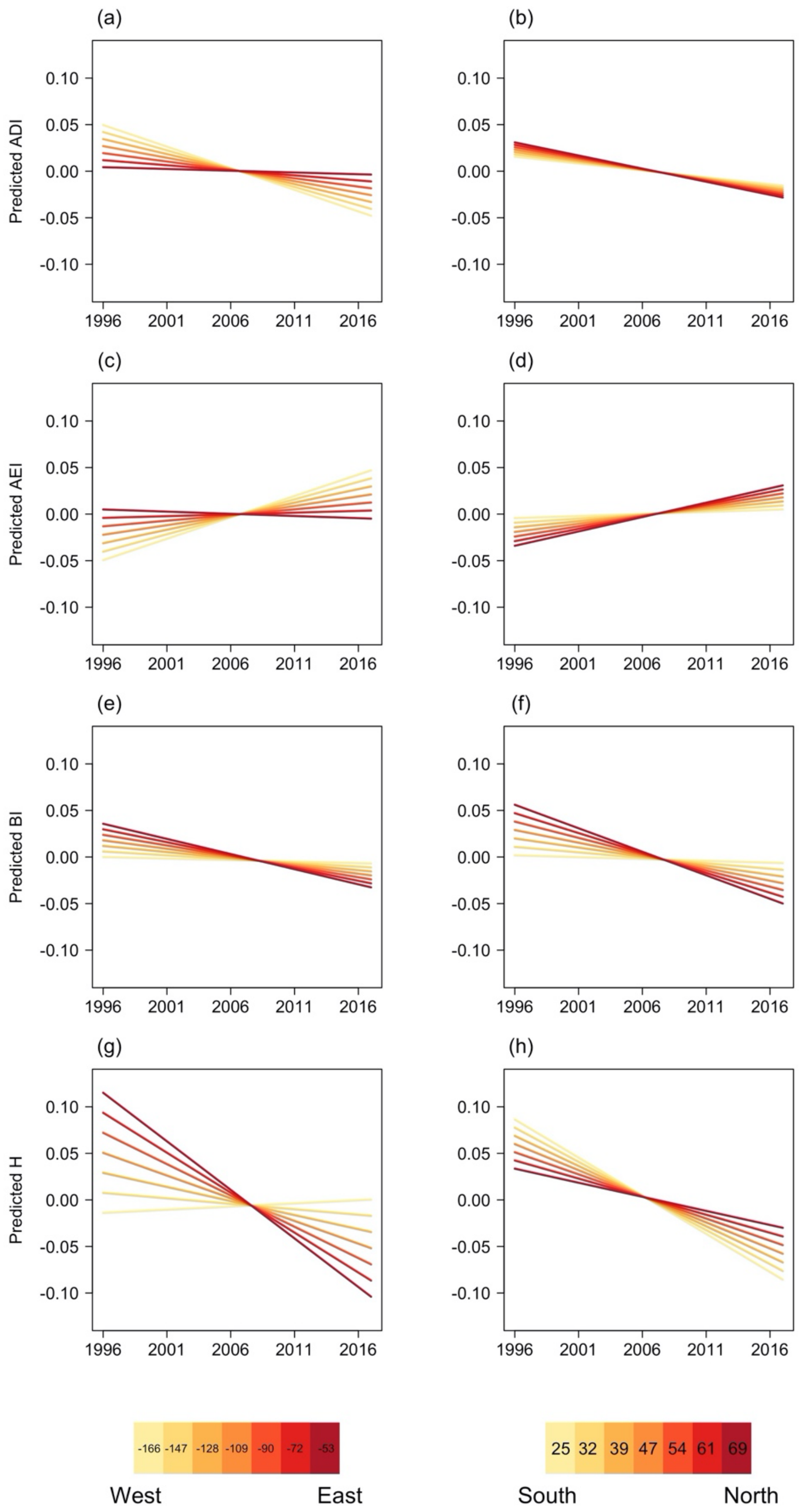

Supplementary Figure 2: Predicted trends in acoustic indices across North America.

Predicted temporal trends in Acoustic Diversity Index, ADI (a,b), Acoustic Evenness Index, AEI (c,d), Bioacoustic Index, BI (e,f) and Acoustic Entropy, H (g,h) in North America between

1996 and 2017. Coloured lines indicate predicted trends (Supplementary Table 1) at varying longitudes (left column) and latitudes (right column) at equal intervals across the range of the dataset from west and south (yellow) to east and north (red). Note that predicted trends reflect temporal changes in standardised acoustic indices.

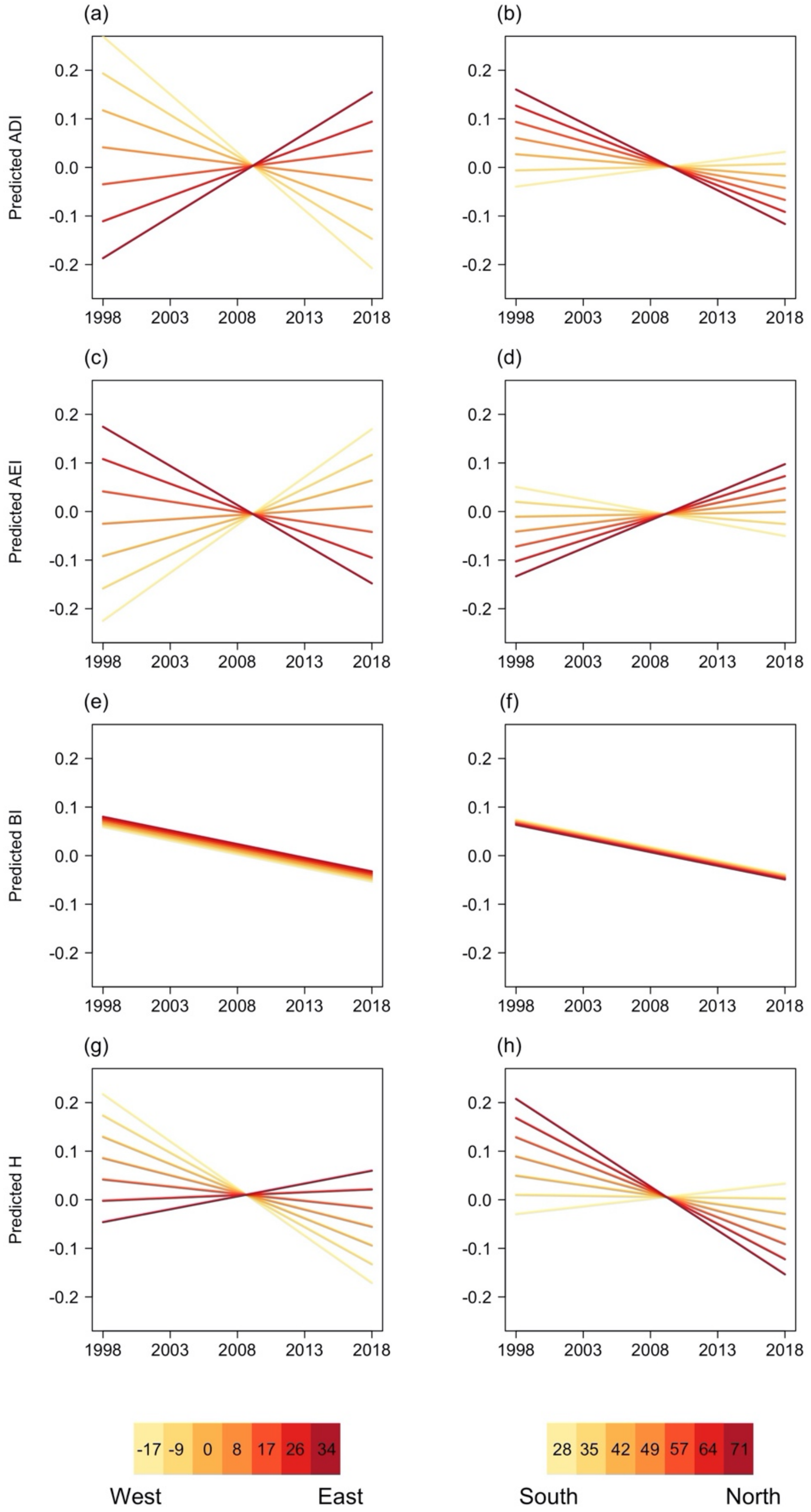

Supplementary Figure 3: Predicted trends in acoustic indices across Europe. Predicted temporal trends in Acoustic Diversity Index, ADI (a,b), Acoustic Evenness Index, AEI (c,d), Bioacoustic Index, BI (e,f) and Acoustic Entropy, H (g,h) in Europe between 1998 and 2018.

Coloured lines indicate predicted trends (Supplementary Table 2) at varying longitudes (left column) and latitudes (right column) at equal intervals across the range of the dataset from west and south (yellow) to east and north (red). Note that predicted trends reflect temporal changes in standardised acoustic indices.

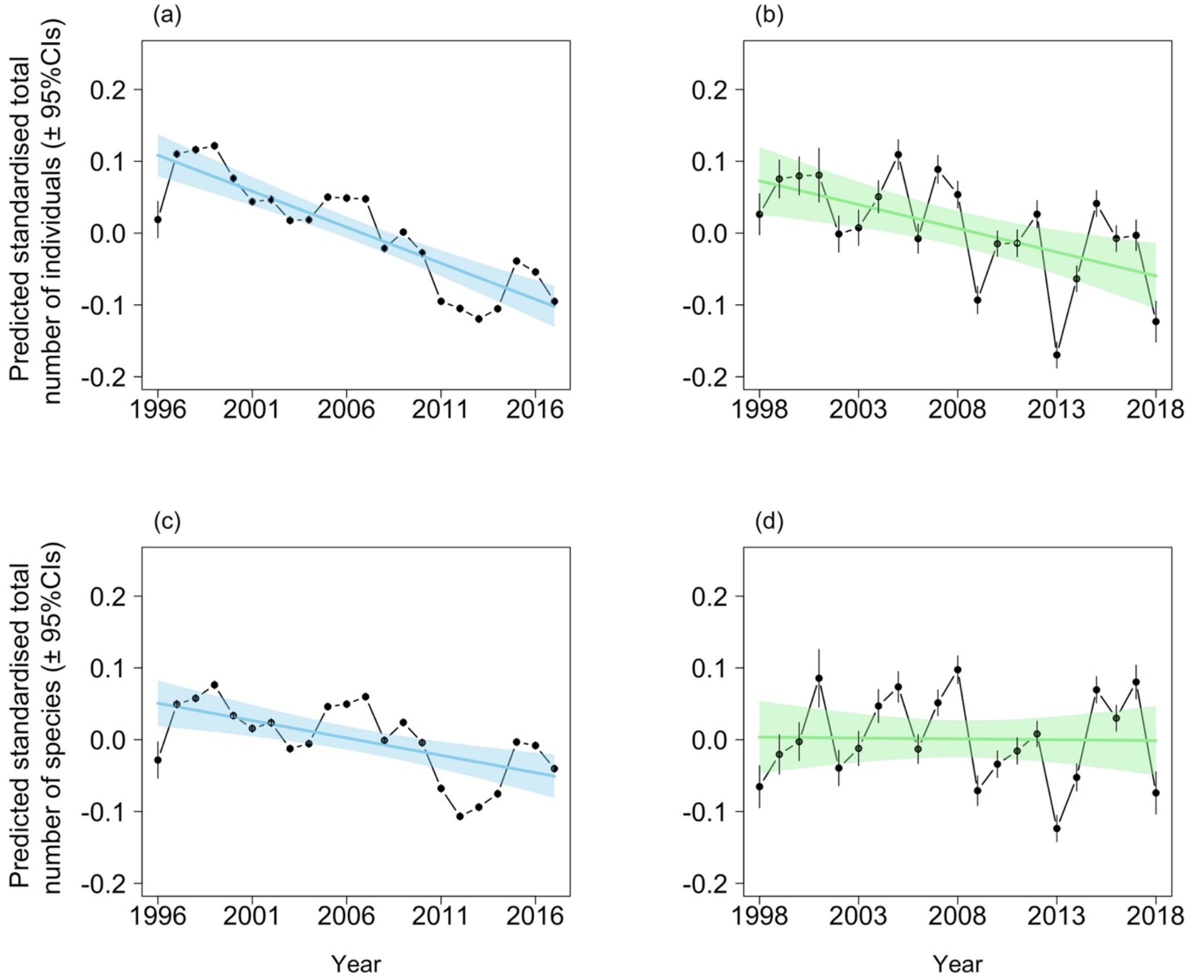

Supplementary Figure 4: Temporal trends in abundance and species richness. Predicted annual variation in the total number of individuals (a,c) and total number of species (b,d) in North America (left column) between 1996 and 2017 and in Europe (right column) between 1998 and 2018. Blue (North America) and green (Europe) lines show the predicted trends from GLMMs (Supplementary Table 4); shaded areas indicate 95% confidence intervals. Points show predicted annual values from GLMMs with the identical structure as those in Supplementary Table 4 but with year fitted as a categorical rather than a continuous variable, vertical lines indicate the 95% confidence intervals. Note that the total number of individuals and species recorded each year at each site were standardised prior to analyses.

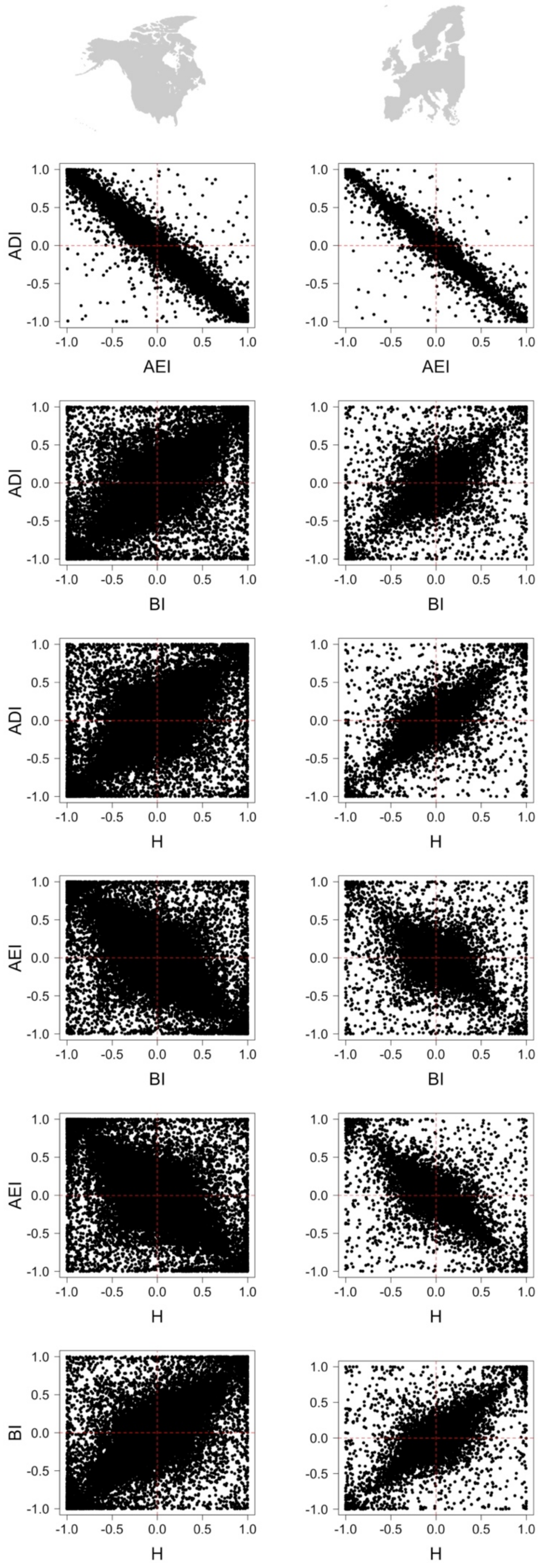
ADI
AEI
ADI
BI
ADI
H
AEI
BI
AEI
H
BI
H

Supplementary Figure 5: Correlations in acoustic indices. Pairwise associations between site-level trends in acoustic indices (Acoustic Diversity Index (ADI), Acoustic Evenness Index (AEI), Bioacoustic Index (BI), Acoustic Entropy (H)) in 202737 NA-BBS sites across North America (left column) and in 16524 PECBMS sites across Europe (right column). Note that site-level trends are derived from changes in standardised annual values of each acoustic index.

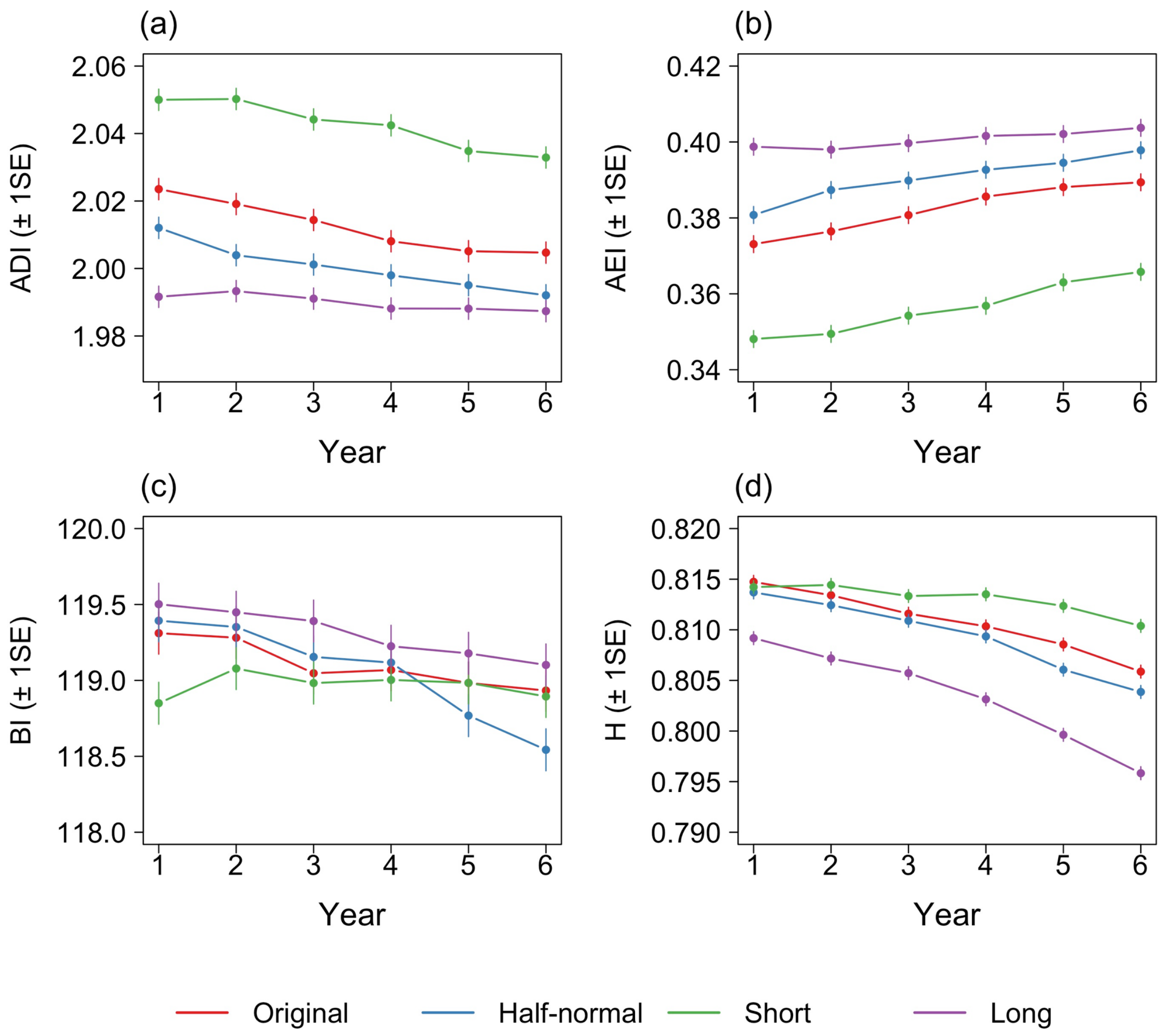

Supplementary Figure 6: Sensitivity of acoustic metrics to soundscape construction methods. Mean (±1 standard error) Acoustic Diversity Index, ADI (a), Acoustic Evenness Index, AEI (b), Bioacoustic Index, BI (c) and Acoustic Entropy, H (d) of constructed soundscapes over a 6-year period for 1000 communities containing 10 randomly sampled European species. The abundance of each species in Year 1 was set to 10 individuals and declined linearly to five individuals by Year 6. Each line represents acoustic index values for soundscapes constructed using different methods: Original – default setting of 5-minute soundscape and playback volume sampled from a uniform distribution; Half-normal: 5-minute soundscape with playback volume sampled from a half-normal distribution; Short: 3-

minute soundscape with playback volume sampled from a uniform distribution; Long: 10-minute soundscape with playback volume sampled from a uniform distribution.

Supplementary Table 1. Results of GLMMs of the variation in a) Acoustic Diversity Index (ADI), (b) Acoustic Evenness Index (AEI), (c) Bioacoustic Index (BI) and (d) Acoustic Entropy (H) in 202737 NA-BBS sites across North America surveyed in at least three years between 1996 and 2017. Note that annual values for each acoustic index were standardised at the site-level prior to analyses.

	**Fixed effects**	**Estimate (SE)**	**χ^2**	**DF**	**p**
(a) ADI	Latitude	0.00038 (0.00020)	0.01	1	0.951
	Longitude	-0.00044 (0.00008)	0.32	1	0.569
	Year	0.00285 (0.00095)	8.46	1	0.004
	Latitude*year	-0.00003 (0.00001)	4.21	1	0.004
	Longitude*year	0.00003 (0.00001)	43.11	1	<0.001
(b) AEI	Latitude	-0.00073 (0.00020)	0.01	1	0.930
	Longitude	0.00052 (0.00007)	0.23	1	0.634
	Year	-0.00528 (0.00087)	6.47	1	0.011
	Latitude*year	0.00006 (0.00001)	16.45	1	<0.001
	Longitude*year	-0.00004 (0.00001)	60.80	1	<0.001
(c) BI	Latitude	0.00134 (0.00020)	0.18	1	0.667
	Longitude	0.00034 (0.00008)	0.68	1	0.409
	Year	-0.00027 (0.00110)	6.08	1	0.014
	Latitude*year	-0.00011 (0.00001)	51.10	1	<0.001
	Longitude*year	-0.00003 (0.00001)	20.36	1	<0.001
(d) H	Latitude	-0.00131 (0.00020)	2.26	1	0.132

Longitude	0.00124 (0.00007)	1.89	1	0.169
Year	-0.02041 (0.00186)	12.79	1	<0.001
Latitude*year	0.00012 (0.00001)	63.03	1	<0.001
Longitude*year	-0.00010 (0.00001)	293.03	1	<0.001

Supplementary Table 2. Results of GLMMs of the variation in a) Acoustic Diversity Index (ADI), (b) Acoustic Evenness Index (AEI), (c) Bioacoustic Index (BI) and (d) Acoustic Entropy (H) in in 16524 PECBMS sites across Europe surveyed in at least three years between 1998 and 2018. Only significant interaction effects are retained. Note that annual values for each acoustic index were standardised at the site-level prior to analyses.

	**Fixed effects**	**Estimate (SE)**	χ^2	**DF**	**p**
(a) ADI	Latitude	0.0054 (0.0013)	0.05	1	0.816
	Longitude	-0.0093 (0.0008)	3.48	1	0.062
	Year	0.0109 (0.0058)	5.99	1	0.014
	Latitude*year	-0.0004 (0.0001)	17.64	1	<0.001
	Longitude*year	0.0008 (0.0001)	206.13	1	<0.001
(b) AEI	Latitude	-0.0046 (0.0013)	0.30	1	0.582
	Longitude	0.0085 (0.0008)	2.72	1	0.099
	Year	-0.0123 (0.0058)	3.09	1	0.079
	Latitude*year	0.0004 (0.0001)	16.00	1	<0.001
	Longitude*year	-0.0007 (0.0001)	158.13	1	<0.001
(c) BI	Latitude	-0.0002 (0.0005)	0.24	1	0.621
	Longitude	0.0004 (0.0003)	2.26	1	0.132
	Year	-0.0056 (0.0021)	6.91	1	0.009
(d) H	Latitude	0.0060 (0.0013)	0.32	1	0.571
	Longitude	-0.0056 (0.0008)	4.07	1	0.044
	Year	0.0146 (0.0059)	10.02	1	0.001

Latitude*year	-0.0005 (0.0001)	26.14	1	<0.001
Longitude*year	0.0005 (0.0001)	75.14	1	<0.001

Supplementary Table 3: Results of GLMMs of the variation in community structure: (a) the total number of individuals and (b) the total number of species in 202737 NA-BBS sites across North America between 1996 and 2017 and in 16524 PECBMS sites across Europe between 1998 and 2018. Note that the total number of individuals and species recorded each year at each site were standardised prior to analyses.

	**Fixed effects**	**North America**				**Europe**			
		**Estimate (SE)**	**χ^2**	**DF**	**p**	**Estimate (SE)**	**χ^2**	**DF**	**p**
(a) Total number of individuals	Latitude	0.0001 (0.0001)	2.80	1	0.094	0.0009 (0.0005)	0.03	1	0.848
	Longitude	0.0001 (0.0001)	7.21	1	0.007	-0.0002 (0.0003)	0.33	1	0.564
	Year	-0.0100 (0.0012)	66.2	1	<0.001	-0.0003 (0.0022)	0.02	1	0.887
(b) Total number of species	Latitude	0.0001 (0.0001)	0.587	1	0.044	0.0001 (0.0005)	0.03	1	0.863
	Longitude	0.0001 (0.0001)	2.030	1	0.154	0.0001 (0.0003)	0.38	1	0.537
	Year	-0.0048 (0.0013)	13.12	1	<0.001	-0.0066 (0.0021)	9.96	1	0.002

Supplementary Table 4: Results of Pearson correlations of pairwise associations between site-level trends in acoustic indices, Acoustic Diversity Index (ADI), Acoustic Evenness Index (AEI), Bioacoustic Index (BI) and Acoustic Entropy (H), in 202737 NA-BBS sites across North America and in 16524 PECBMS sites across Europe. Note that site-level trends are derived from changes in standardised annual values of each acoustic index.

	**North America**			**Europe**		
Pairwise comparison	**r**	**t**	**p**	**r**	**t**	**p**
ADI-AEI	-0.97	-1921	<0.001	-0.95	-385.07	<0.001
ADI-BI	0.59	329.53	<0.001	0.47	67.82	<0.001
ADI-H	0.54	288.38	<0.001	0.62	102.00	<0.001
AEI-BI	-0.56	-307.65	<0.001	-0.40	-54.98	<0.001
AEI-H	-0.51	-271.81	<0.001	-0.61	-100.14	<0.001
BI-H	0.67	404.23	<0.001	0.62	101.47	<0.001

Supplementary Table 5: The survey method, number of sites included in our analyses and period covered by each of the PECBMS schemes.

**Country**	**Survey method**	**Number of sites**	**Range of time series**
Austria	Point counts	348	1998 – 2017
Belgium	Point counts	252	1998 – 2017
Bulgaria	Line transects	176	2005 – 2016
Catalonia	Line transects	327	2002 – 2016
Cyprus	Line transects	136	2006 - 2017
Czech Republic	Point counts	197	1998 - 2018
Denmark	Point counts	708	1998 - 2016
Estonia	Point counts	15	1998 - 2016
Finland	Line transects and point counts	706	1998 - 2016
France	Point counts	1484	2001 - 2017
Germany	Line transects	1324	2005 - 2016
Greece	Point counts	44	2007 - 2016
Netherlands	Territory mapping	1510	1998 - 2016
Ireland	Line transects	399	1998 - 2016
Latvia	Line transects	45	2005 - 2016
Norway	Point counts	411	2006 - 2018
Poland	Line transects	975	2000 - 2017
Romania	Point counts	200	2007 - 2017
Slovenia	Line transects	104	2007 - 2017

Spain	Point counts	721	1998 - 2016
Sweden	Line transects and point counts	716	1998 - 2017
Switzerland	Territory mapping	259	1999 - 2016
United Kingdom	Line transects	5467	1998 – 2018

Supplementary Table 6: Results of Moran's I test for spatial autocorrelation in the modelled residuals of the GLMMs for North America (Supplementary Table 1) and Europe (Supplementary Table 2): a) Acoustic Diversity Index (ADI), (b) Acoustic Evenness Index (AEI), (c) Bioacoustic Index (BI) and (d) Acoustic Entropy (H). Years were tested separately.

	North America					Europe				
	**Year**	**Observed**	**Expected**	**Standard deviation**	**p**	**Year**	**Observed**	**Expected**	**Standard deviation**	**p**
(a) ADI	1996	-0.0492	-0.0085	0.0278	0.1428	1998	0.0038	-0.0002	0.0012	0.0010
	1997	0.0006	-0.0004	0.0011	0.3691	1999	0.0075	-0.0002	0.0011	<0.001
	1998	0.0026	-0.0004	0.0012	0.0116	2000	0.0072	-0.0002	0.0011	<0.001
	1999	0.0017	-0.0004	0.0013	0.1105	2001	0.0109	-0.0004	0.0028	<0.001
	2000	0.0007	-0.0004	0.0013	0.4134	2002	0.0054	-0.0002	0.0010	<0.001
	2001	0.0000	-0.0004	0.0013	0.7600	2003	0.0082	-0.0002	0.0009	<0.001
	2002	-0.0016	-0.0004	0.0014	0.3895	2004	0.0053	-0.0002	0.0008	<0.001
	2003	0.0007	-0.0004	0.0014	0.4301	2005	0.0022	-0.0001	0.0007	<0.001
	2004	0.0020	-0.0004	0.0011	0.0234	2006	0.0025	-0.0001	0.0006	<0.001
	2005	0.0006	-0.0004	0.0011	0.4020	2007	0.0049	-0.0001	0.0006	<0.001
	2006	0.0022	-0.0004	0.0013	0.0468	2008	0.0026	-0.0001	0.0006	<0.001
	2007	0.0024	-0.0004	0.0011	0.0098	2009	0.0031	-0.0001	0.0006	<0.001
	2008	0.0010	-0.0004	0.0014	0.3321	2010	0.0019	-0.0001	0.0005	<0.001
	2009	0.0004	-0.0004	0.0012	0.5331	2011	0.0064	-0.0001	0.0005	<0.001
	2010	-0.0013	-0.0004	0.0014	0.5020	2012	0.0035	-0.0001	0.0004	<0.001
	2011	0.0032	-0.0004	0.0010	<0.001	2013	0.0031	-0.0001	0.0004	<0.001

	2012	0.0006	-0.0004	0.0014	0.4532	2014	0.0022	-0.0001	0.0005	<0.001
	2013	-0.0005	-0.0004	0.0012	0.9273	2015	0.0026	-0.0001	0.0004	<0.001
	2014	-0.0001	-0.0004	0.0015	0.8354	2016	0.0045	-0.0001	0.0005	<0.001
	2015	0.0025	-0.0004	0.0014	0.0355	2017	0.0039	-0.0002	0.0006	<0.001
	2016	0.0011	-0.0004	0.0012	0.2266	2018	0.0023	-0.0003	0.0007	<0.001
	2017	0.0008	-0.0004	0.0013	0.3554					
**(b) AEI**	1996	-0.0424	-0.0085	0.0278	0.2220	1998	0.0043	-0.0002	0.0012	<0.001
	1997	0.0006	-0.0004	0.0011	0.3949	1999	0.0060	-0.0002	0.0011	<0.001
	1998	0.0025	-0.0004	0.0012	0.0134	2000	0.0059	-0.0002	0.0011	<0.001
	1999	0.0015	-0.0004	0.0013	0.1406	2001	0.0122	-0.0004	0.0028	<0.001
	2000	0.0006	-0.0004	0.0013	0.4438	2002	0.0058	-0.0002	0.0010	<0.001
	2001	0.0002	-0.0004	0.0013	0.6642	2003	0.0080	-0.0002	0.0009	<0.001
	2002	-0.0019	-0.0004	0.0014	0.2764	2004	0.0058	-0.0002	0.0008	<0.001
	2003	0.0021	-0.0004	0.0014	0.0747	2005	0.0019	-0.0001	0.0007	<0.001
	2004	0.0020	-0.0004	0.0011	0.0235	2006	0.0024	-0.0001	0.0006	<0.001
	2005	-0.0001	-0.0004	0.0011	0.7860	2007	0.0048	-0.0001	0.0006	<0.001
	2006	0.0020	-0.0004	0.0013	0.0659	2008	0.0028	-0.0001	0.0006	<0.001
	2007	0.0023	-0.0004	0.0011	0.0121	2009	0.0036	-0.0001	0.0006	<0.001
	2008	0.0020	-0.0004	0.0014	0.0900	2010	0.0022	-0.0001	0.0005	<0.001
	2009	0.0000	-0.0004	0.0012	0.7650	2011	0.0079	-0.0001	0.0005	<0.001
	2010	-0.0018	-0.0004	0.0014	0.3101	2012	0.0034	-0.0001	0.0004	<0.001
	2011	0.0028	-0.0004	0.0010	0.0016	2013	0.0036	-0.0001	0.0004	<0.001
	2012	0.0004	-0.0004	0.0014	0.5842	2014	0.0016	-0.0001	0.0005	<0.001

	2013	-0.0001	-0.0004	0.0012	0.7947	2015	0.0024	-0.0001	0.0004	<0.001
	2014	0.0002	-0.0004	0.0015	0.6865	2016	0.0056	-0.0001	0.0005	<0.001
	2015	0.0020	-0.0004	0.0014	0.0799	2017	0.0032	-0.0002	0.0006	<0.001
	2016	0.0016	-0.0004	0.0012	0.1135	2018	0.0026	-0.0003	0.0007	<0.001
	2017	0.0014	-0.0004	0.0013	0.1684					
**(c) BI**	1996	-0.0017	-0.0085	0.0278	0.8068	1998	0.0078	-0.0002	0.0012	<0.001
	1997	0.0019	-0.0004	0.0011	0.0465	1999	0.0078	-0.0002	0.0011	<0.001
	1998	0.0033	-0.0004	0.0012	0.0017	2000	0.0047	-0.0002	0.0011	<0.001
	1999	0.0006	-0.0004	0.0013	0.4415	2001	0.0145	-0.0004	0.0028	<0.001
	2000	-0.0003	-0.0004	0.0013	0.9230	2002	0.0013	-0.0002	0.0010	0.1405
	2001	0.0015	-0.0004	0.0013	0.1552	2003	0.0056	-0.0002	0.0009	<0.001
	2002	-0.0002	-0.0004	0.0014	0.8886	2004	0.0031	-0.0002	0.0008	<0.001
	2003	0.0000	-0.0004	0.0014	0.7950	2005	0.0024	-0.0001	0.0007	<0.001
	2004	0.0009	-0.0004	0.0011	0.2110	2006	0.0012	-0.0001	0.0006	0.0371
	2005	0.0006	-0.0004	0.0011	0.3857	2007	0.0023	-0.0001	0.0006	<0.001
	2006	0.0009	-0.0004	0.0013	0.3178	2008	0.0024	-0.0001	0.0006	<0.001
	2007	0.0027	-0.0004	0.0011	0.0043	2009	0.0020	-0.0001	0.0006	<0.001
	2008	0.0020	-0.0004	0.0014	0.0951	2010	0.0025	-0.0001	0.0005	<0.001
	2009	-0.0015	-0.0004	0.0012	0.3652	2011	0.0008	-0.0001	0.0005	0.0716
	2010	-0.0018	-0.0004	0.0014	0.3241	2012	0.0023	-0.0001	0.0004	<0.001
	2011	0.0012	-0.0004	0.0010	0.1123	2013	0.0021	-0.0001	0.0004	<0.001
	2012	0.0015	-0.0004	0.0014	0.1568	2014	0.0016	-0.0001	0.0005	<0.001
	2013	-0.0004	-0.0004	0.0012	0.9789	2015	0.0033	-0.0001	0.0004	<0.001

	2014	-0.0020	-0.0004	0.0015	0.2761	2016	0.0072	-0.0001	0.0005	<0.001
	2015	0.0020	-0.0004	0.0014	0.0814	2017	0.0043	-0.0002	0.0006	<0.001
	2016	0.0018	-0.0004	0.0012	0.0748	2018	0.0014	-0.0003	0.0007	0.0255
	2017	0.0000	-0.0004	0.0013	0.7756					
**(d) H**	1996	0.0197	-0.0085	0.0279	0.3119	1998	0.0133	-0.0002	0.0012	<0.001
	1997	0.0025	-0.0004	0.0011	0.0093	1999	0.0131	-0.0002	0.0011	<0.001
	1998	0.0028	-0.0004	0.0012	0.0072	2000	0.0111	-0.0002	0.0011	<0.001
	1999	0.0018	-0.0004	0.0013	0.0884	2001	0.0193	-0.0004	0.0028	<0.001
	2000	0.0005	-0.0004	0.0013	0.5046	2002	0.0074	-0.0002	0.0010	<0.001
	2001	0.0021	-0.0004	0.0013	0.0599	2003	0.0092	-0.0002	0.0009	<0.001
	2002	0.0023	-0.0004	0.0014	0.0544	2004	0.0067	-0.0002	0.0008	<0.001
	2003	0.0003	-0.0004	0.0014	0.6037	2005	0.0034	-0.0001	0.0007	<0.001
	2004	0.0023	-0.0004	0.0011	0.0112	2006	0.0037	-0.0001	0.0006	<0.001
	2005	0.0000	-0.0004	0.0011	0.7559	2007	0.0042	-0.0001	0.0006	<0.001
	2006	0.0004	-0.0004	0.0013	0.5399	2008	0.0026	-0.0001	0.0006	<0.001
	2007	0.0044	-0.0004	0.0011	<0.001	2009	0.0061	-0.0001	0.0006	<0.001
	2008	0.0035	-0.0004	0.0014	0.0058	2010	0.0039	-0.0001	0.0005	<0.001
	2009	0.0015	-0.0004	0.0012	0.1263	2011	0.0028	-0.0001	0.0005	<0.001
	2010	0.0005	-0.0004	0.0014	0.5455	2012	0.0017	-0.0001	0.0004	<0.001
	2011	0.0039	-0.0004	0.0010	<0.001	2013	0.0048	-0.0001	0.0004	<0.001
	2012	0.0026	-0.0004	0.0014	0.0286	2014	0.0051	-0.0001	0.0005	<0.001
	2013	0.0018	-0.0004	0.0012	0.0719	2015	0.0070	-0.0001	0.0004	<0.001
	2014	-0.0011	-0.0004	0.0015	0.6392	2016	0.0145	-0.0001	0.0005	<0.001

2015	0.0042	-0.0004	0.0014	<0.001	2017	0.0071	-0.0002	0.0006	<0.001
2016	0.0007	-0.0004	0.0012	0.3899	2018	0.0021	-0.0003	0.0007	0.0017
2017	-0.0015	-0.0004	0.0013	0.3591					
