# Supplementary material for: Bird population declines and species turnover are changing the acoustic properties of spring soundscapes
Source: Nat Commun. 2021 Nov 2;12:6217. doi: 10.1038/s41467-021-26488-1 (PMC8564540; doi:10.1038/s41467-021-26488-1)
Supplement: Supplementary file 3 — Reporting Summary [file 41467_2021_26488_MOESM3_ESM.pdf]

nature research

Corresponding author(s): Simon Butler

Last updated by author(s): 07/9/2021

Reporting Summary

Nature Research wishes to improve the reproducibility of the work that we publish. This form provides structure for consistency and transparency in reporting. For further information on Nature Research policies, see Authors & Referees and the Editorial Policy Checklist .

Statistics

For all statistical analyses, confirm that the following items are present in the figure legend, table legend, main text, or Methods section.

n/a	Confirmed	
☐	☒	The exact sample size (n) for each experimental group/condition, given as a discrete number and unit of measurement
☐	☒	A statement on whether measurements were taken from distinct samples or whether the same sample was measured repeatedly
☐	☒	The statistical test(s) used AND whether they are one- or two-sided<br>*Only common tests should be described solely by name; describe more complex techniques in the Methods section.*
☐	☒	A description of all covariates tested
☐	☒	A description of any assumptions or corrections, such as tests of normality and adjustment for multiple comparisons
☐	☒	A full description of the statistical parameters including central tendency (e.g. means) or other basic estimates (e.g. regression coefficient) AND variation (e.g. standard deviation) or associated estimates of uncertainty (e.g. confidence intervals)
☐	☒	For null hypothesis testing, the test statistic (e.g. F, t, r) with confidence intervals, effect sizes, degrees of freedom and P value noted<br>*Give P values as exact values whenever suitable.*
☒	☐	For Bayesian analysis, information on the choice of priors and Markov chain Monte Carlo settings
☒	☐	For hierarchical and complex designs, identification of the appropriate level for tests and full reporting of outcomes
☐	☒	Estimates of effect sizes (e.g. Cohen's d, Pearson's r), indicating how they were calculated

Our web collection on statistics for biologists contains articles on many of the points above.

Software and code

Policy information about availability of computer code

Data collection	Soundscapes were reconstructed using Sound eXchange (http://sox.sourceforge.net/) v14.4.2
Data analysis	All data analysis was carried out in R version 3.5.1. R packages used: ape (v5.4-1), lme4 (v1.1-25), seewave (v2.1.0), soundecology (v1.3.3), tuneR (v1.3.3). R code for soundscape construction, extraction of acoustic indices and statistical analyses are available from the Open Science Framework under accession code: https://osf.io/jyuxk/

For manuscripts utilizing custom algorithms or software that are central to the research but not yet described in published literature, software must be made available to editors/reviewers. We strongly encourage code deposition in a community repository (e.g. GitHub). See the Nature Research guidelines for submitting code & software for further information.

Data

Policy information about availability of data

All manuscripts must include a data availability statement. This statement should provide the following information, where applicable:

- Accession codes, unique identifiers, or web links for publicly available datasets
- A list of figures that have associated raw data
- A description of any restrictions on data availability

The North American bird monitoring data are available directly from U.S. Geological Survey (https://www.pwrc.usgs.gov/bbs/) and the European bird monitoring are available, on request, from PECBMS (https://pecbms.info/). Sound recordings were downloaded from Xeno Canto (http://www.xeno-canto.org). Acoustic indices for soundscapes constructed from simulated communities, site-level acoustic index data for reconstructed soundscapes for NA-BBS and PECBMS sites, and source data for all figures are available from the Open Science Framework under accession code: https://osf.io/jyuxk/

Field-specific reporting

Please select the one below that is the best fit for your research. If you are not sure, read the appropriate sections before making your selection.

☐ Life sciences ☐ Behavioural & social sciences ☒ Ecological, evolutionary & environmental sciences

For a reference copy of the document with all sections, see nature.com/documents/nr-reporting-summary-flat.pdf

Ecological, evolutionary & environmental sciences study design

All studies must disclose on these points even when the disclosure is negative.

Study description

The study integrates citizen science bird monitoring data (North American Breeding Bird Survey; Pan-European Common Bird Monitoring Scheme) with recordings of individual species (Xeno Canto) to reconstruct soundscapes at more than 200,000 sites across North America and Europe over the past 25 years. We use four acoustic metrics (Acoustic Diversity Index; Acoustic Evenness Index; Bioacoustic Index; Acoustic Entropy) to quantify soundscape characteristics and estimate site-level trends in each.

Research sample

We report changes in soundscape characteristics across North America and Europe since 1998. We use data from large-scale, citizen science monitoring schemes designed to monitor the status and trends of birds across each continent. Specifically, we use i) North American Breeding Bird Survey data (NA-BBS: https://www.pwrc.usgs.gov/bbs/) to estimate site-level trends in Acoustic Diversity Index; Acoustic Evenness Index; Bioacoustic Index; Acoustic Entropy of reconstructed soundscapes at 202737 sites between 1996 and 2017 and ii) Pan-European Common Bird Monitoring Scheme data (PECBMS: https://pecbms.info/) to estimate site-level trends in Acoustic Diversity Index; Acoustic Evenness Index; Bioacoustic Index; Acoustic Entropy of reconstructed soundscapes at 16524 sites between 1998 and 2018. We use the same bird monitoring data to calculate trends in total abundance and species richness at each site.

Sampling strategy

Bird count data: The North American Breeding Bird Survey (NA-BBS) and Pan-European Common Bird Monitoring Scheme (PECBMS) are specifically designed to monitor the status and trends of birds across each continent. NA-BBS survey routes, consisting of 50 survey points evenly distributed over approximately 24.5 miles, are distributed across the United States and Canada and are usually surveyed in June. At each site, skilled volunteers conduct a three-minute point count, recording all birds seen or heard within a 400-meter radius. PECBMS collates monitoring data from 23 survey schemes across 22 countries. In each scheme, skilled volunteers carry out either line transects, point counts or territory mapping at survey sites during the breeding season and record all birds encountered; whilst methods vary between survey schemes, they are consistent within schemes across the time period included here. Not all sites are surveyed in every year and only data from sites surveyed at least three times during the defined time period were included in analyses. Similar results were found when restricting data to sites surveyed in at least 10 years during the defined period.

Sound recordings: Sound files for all species detected on NA-BBS and PECBMS surveys were downloaded from http://www.xeno-canto.org. For a given species, we identified all files longer than 30s, with associated metadata categorising them as high quality (category "A") and as either "song", "call" or "drumming" types; sound files whose type category including the term "wingbeat", "flap", "begging", "alarm" or "night" types were excluded. Sound files downloaded for NA-BBS species were restricted to those recorded in North America and those from PECBMS to recordings made in Europe. If no sound files met these requirements for a given species, we downloaded all files of shorter duration for that species that met the quality and type criteria and stitched repeats of these together to produce files longer than 30s. If no sound files for a species were available, the sites where that species was detected were removed from subsequent analyses. Each downloaded sound file was then standardised to ensure consistent sampling rate, duration and volume. Where more than 50 sound files were available for a species, a random selection of 50 was taken for use in subsequent analyses.

Data collection

The data used were previously collected by several thousand skilled citizen scientists over ~20 years, using standardised survey techniques to record all birds seen or heard within the survey area - defined in this paper as a "site". Full details of the data collection methodologies for country-specific monitoring schemes are available at: https://pecbms.info/ and https://www.pwrc.usgs.gov/bbs/.

Timing and spatial scale

North American Breeding Bird Survey: We use data from annual surveys of abundance between 1996 and 2017 at 202737 sites across North America. Each site is a survey point along a NA-BBS route. Survey points are evenly distributed over routes of approximately 24.5 miles, which are distributed across the United States and Canada; we include data from 4196 survey routes in our analyses. PECBMS data: We use data from annual surveys of abundance between 1998 and 2018 at 16524 sites from 23 survey schemes in 22 countries. Data cover an approximately 20 year time period that allows broad-scale analysis of changes in soundscape characteristics across the two continents and includes the latest site-level data available at the point the research was initiated.

Data exclusions

Only sites that had been surveyed at least three times during the defined time period (NA-BBS: 1996-2017; PECBMS: 1998-2018) were included in analyses. If no sound files for a species were available on Xeno-Canto (see Sampling strategy), the sites where that species was detected were removed from subsequent analyses; this represented <1.5% NA-BBS sites and <3.5% PECBMS sites.

Reproducibility

Count data collected at 202737 sites in North America between 1996 and 2017 and 16524 sites in Europe between 1998 and 2018 were used for soundscape construction. The soundscape construction process involves random sampling of species' sound files as well as a randomised allocation of playback volume and soundscape insertion point. The acoustic properties of the soundscape constructed for a given community will therefore differ on each iteration. We use the average of five iterations in subsequent analyses of changes in soundscape characteristics across North America and Europe. These data, and R codes for soundscape reconstruction and data analyses, are available from the Open Science Framework under accession code: https://osf.io/jyuxk/

Randomization	Where more than 50 sound files were available for a species, a random selection of 50 was taken for use in subsequent analyses. Soundscape reconstructions were iterated five times for each site-year combination due to i) randomised selection of sound files for given species, ii) randomised allocation of playback volume and iii) randomised soundscape insertion point, with each acoustic metric averaged across these five site-year iterations for use in subsequent analyses.
Blinding	This is a non-experimental study so blinding is not applicable.

Did the study involve field work? ☐ Yes ☒ No

Reporting for specific materials, systems and methods

We require information from authors about some types of materials, experimental systems and methods used in many studies. Here, indicate whether each material, system or method listed is relevant to your study. If you are not sure if a list item applies to your research, read the appropriate section before selecting a response.

Materials & experimental systems

n/a	Involved in the study	
☒	☐	Antibodies
☒	☐	Eukaryotic cell lines
☒	☐	Palaeontology
☒	☐	Animals and other organisms
☒	☐	Human research participants
☒	☐	Clinical data

Methods

n/a	Involved in the study	
☒	☐	ChIP-seq
☒	☐	Flow cytometry
☒	☐	MRI-based neuroimaging
